# Four-decade trends in lymph node status of patients with vulvar squamous cell carcinoma in northern Italy

**DOI:** 10.1038/s41598-021-85030-x

**Published:** 2021-03-11

**Authors:** Mario Preti, Lauro Bucchi, Leonardo Micheletti, Silvana Privitera, Monica Corazza, Stefano Cosma, Niccolò Gallio, Alessandro Borghi, Federica Bevilacqua, Chiara Benedetto

**Affiliations:** 1 Department of Surgical Sciences, University of Torino, Torino, Italy; 2 Romagna Cancer Registry, Romagna Cancer Institute (IRCCS Istituto Romagnolo Per Lo Studio Dei Tumori (IRST) “Dino Amadori”), Meldola, Forlì Italy; 3 Department of Pathology, Azienda Ospedaliero-Universitaria (AOU) Città della Salute e della Scienza, Torino, Italy; 4 Department of Medical Sciences, Section of Dermatology and Infectious Diseases, University of Ferrara, Ferrara, Italy

**Keywords:** Cancer, Oncology, Risk factors

## Abstract

The 4-decade (1980–2017) trends in lymph node status of patients with vulvar squamous cell carcinoma (VSCC) in a province of northern Italy were investigated. Information was collected on lymph node dissection, number of lymph nodes dissected, lymph node involvement, and number of positive lymph nodes from a series of 760 patients admitted to a tertiary referral centre for vulvar disease. The adjusted odds ratios (ORs) for lymph node involvement, for ≥ 2 positive nodes, and for a lymph node ratio ≥ 20% were estimated from multiple logistic regression models. The adjusted OR for lymph node dissection was greater in the 2000s and 2010s versus the 1980s. The adjusted OR for lymph node involvement was 1.36 (95% confidence interval (CI), 0.72–2.60) in the 1990s, 1.31 (95% CI, 0.72–2.38) in the 2000s and 1.32 (95% CI, 0.73–2.41) in the 2010s versus the 1980s. The adjusted OR for ≥ 2 positive nodes was 1.36 (95% CI, 0.68–2.72), 0.86 (95% CI, 0.44–1.65) and 0.67 (95% CI, 0.34–1.31), respectively. The adjusted OR for lymph node ratio ≥ 20% was 1.45 (95% CI, 0.62–3.43), 1.21 (95% CI, 0.54–2.72) and 0.81 (95% CI, 0.35–1.89), respectively. This stagnation indicates the need for a serious rethink of the local model for the care of VSCC.

## Introduction

According to many studies from the Western countries, progresses in the outcome of patients with vulvar squamous cell carcinoma (VSCC) have seldom been observed over the last decades^1–3^. In the Nordic Countries, for example, only negligible changes in survival rates have occurred during the period 1964–2003^3^. Between 1989–2010, 5-year relative survival of Dutch patients has not improved^2^. In the United States, overall survival has stagnated from 1988 to 2007^1^.

There are multiple causes for this situation, including, among others, difficulties in carrying out treatment trials and lack of interest by the pharmaceutical industry in developing effective therapies for rare diseases. However, it is likely that the inability to detect VSCC at an earlier stage is another key reason why survival has not increased.

This hypothesis, however, is insufficiently demonstrated. Tumour stage is not routinely recorded in many cancer registries both in Europe and elsewhere^4,5^ and, when available, trend data are often inconsistent. In the Netherlands and Norway, for example, fewer patients have been diagnosed with localised disease in the most recent birth cohorts^2,6^. In Denmark and Germany, conversely, the proportion of patients with small-sized and localised disease has increased over time^7,8^. In the United Kingdom, a trend towards earlier tumour stage has been reported in younger patients but with an opposite trend in older ones^9^. In the United States, stage distribution has remained unchanged for decades^1^.

Even more disquieting is the situation in southern Europe, where problems with the availability of tumour stage data are generalised. In Italy, rare cancers –including vulvar cancer– are omitted from the standard national epidemiologic reports^10^. A monograph dedicated to rare cancers –including vulvar cancer– has reported incidence, survival, and prevalence rates but not tumour stage data nor time trends^11^.

In this article, we report a study of 4-decade trends in lymph node status of patients with VSCC in northern Italy. The study was based on the clinical case records of admissions to a comprehensive tertiary referral centre for vulvar disease. The endpoints included: (1) the prevalence of patients with lymph node involvement, the strongest prognostic factor for VSCC^12–18^ and (2) the distribution by number of positive nodes, one of the most important independent predictors of clinical outcome^19–23^.

## Methods

### Rationale

The databases of tertiary referral centres form the basis of several national^24^ and international^25–27^ registries of rare and understudied malignancies, including VSCC^28^, all over the world. Particularly in southern Europe, under the conditions described above, archival clinical records stored in tertiary referral centres represent irreplaceable research resources^29^. Tertiary referral centres receive patients from all community hospitals and these, in turn, from a number of public and private offices. This referral system lowers the risk of patient selection^27^.

### Setting

This study belongs to a broader systematic investigation on VSCC. The project includes nationwide incidence and survival studies as well as high-resolution studies on diagnosis and treatment of the disease at two tertiary referral centres for vulvar disease, one situated in the Department of Medical Sciences of the University of Ferrara and the other in the St. Anna Hospital, i.e., the section of Obstetrics and Gynaecology of the Department of Surgical Sciences of the University of Torino.

This study was performed at the latter institution. The catchment area of the St. Anna Hospital is the province of Torino, which currently has a female population of 1.172.000. Patients are referred from all gynaecology offices and 12 gynaecology departments of the community hospitals. According to an estimate from the Piedmont Cancer Registry, the St. Anna Hospital sees 59% of incident VSCC cases in the province. The clinical protocols in use are described in other papers^30^. Of note for this study, the implementation of the sentinel lymph node biopsy has been submitted to the Institutional Review Board but the approval is still pending. So far, sentinel lymph node biopsy without complete lymph node dissection has never been performed. Lymph nodes were assessed for metastatic disease using ultrastaging with the hematoxylin and eosin (H&E) staining for the whole study duration. Over the same time period, no patients underwent neoadjuvant therapy.

### Data

Trained personnel retrospectively reviewed the records of VSCC patients who were diagnosed up until 31 December 2017. The data used for the present study were recorded at the time of patient admission and at primary treatment. In case of multiple diagnoses, the index lesion was selected. Seven hundred and ninety-nine consecutive VSCC patients were identified. Twenty-four patients diagnosed during the 1970s and 15 with missing information for the dependent variables were excluded leaving 760 patients eligible for the study.

### Objective

The primary objective of the study was to determine whether any time trend in lymph node involvement and number of positive nodes had occurred among patients presenting at our institution in the 4-decade period between 1980 and 2017. Preliminarily, we evaluated the time trend in the proportion of patients undergoing lymph node dissection and in the number of nodes dissected.

### Design

The time trends in lymph node involvement and number of positive nodes were evaluated among patients undergoing lymph node dissection.

The association of time period with the likelihood of lymph node involvement was adjusted for the following potential confounding factors: patient age^15,17^, tumour size^12^, and depth of stromal invasion^31^, all reported to be strong independent determinants of the risk of lymph node metastasis^18^; disease location, because of the increasing incidence of clitoral VSCC^32^ and its association with more frequent spread to lymph nodes^33^; and number of nodes dissected^34,35^.

The association of time period with the number of positive lymph nodes was assessed with a sensitivity analysis. Three different approaches were used to make allowance for the variation in the number of nodes dissected: (1) adjusting the association for the number of nodes dissected classified as ≤ 10 or > 10, a cut-off value of strong prognostic significance^34,35^; (2) building a second model after exclusion of patients with ≤ 10 nodes dissected; and (3) using the lymph node ratio (LNR), defined as the number of positive nodes divided by the total number of nodes dissected^28,36^, as a third end-point.

### Data analysis

The independent variable –the calendar year– was categorised into decades (1980s, 1990s, 2000s, 2010s), as this was assumed to be the most objective way to treat the variable. With respect to the dependent variables, we used the following criteria: the lymph node involvement was categorised as absent or present (no, yes); the number of positive nodes was categorised as 0–1 or ≥ 2^20^ and 0–2 or ≥ 3^19,21^, with two separate multivariate analyses being performed; and the LNR was categorised into < 20% or ≥ 20%, since patients with the latter characteristics are at increased risk of relapse and cancer-related death^36^.

With respect to the adjusting variables, patient age was treated as a continuous variable. For categorising tumour size, we adopted the cut-off value of 2 cm (≤ 2, > 2) according to the FIGO staging^37^. For the depth of stromal invasion, we used the cut-off value of 5 mm (≤ 5, > 5) for its strong association with overall survival^31^. Disease location was categorised as clitoral or other. The categorisation of the number of lymph nodes dissected and the related criteria are provided in the above Design section.

Differences in proportions were assessed with the chi-square test for trend, and differences in distribution with the Kruskal–Wallis test. The level of statistical significance was set at *P* < 0.05. Multivariate analysis was performed using multiple logistic regression models adjusted for the abovementioned potential confounding factors. All variables were forced into the models. In all models, statistical significance was set at *P* < 0.10. *P* values > 0.05 and < 0.10 were considered to indicate a borderline level of significance.

### Ethics issues

The study was approved by the Ethics Committee at the IRST (ID: IRST100.37). The Ethics Committee waived the requirement of informed consent form for this study due to its retrospective nature and because the analysis was an audit using anonymous and routinely collected clinical data. The study was conducted following the principles of the Declaration of Helsinki and subsequent updates.

## Results

### Clinical characteristics of patients

The number of eligible patients was 116 in the 1980s, 182 in the 1990s, 241 in the 2000s, and 221 in the 2010s. Over the four decades of the study, the median patient age increased from 68 years in the 1980s to 72 in the 1990s, 75 in the 2000s, and 74 in the 2010s (*P* = 0.000). Overall, the age range was 27–101 years.

The population ageing was associated with a significant parallel increase in tumour size. Over the four decades, the proportion of patients with lesions > 2 cm in size was 50.0%, 55.5%, 63.5% and 62.0% (*P* = 0.017), respectively. In multiple logistic regression analysis, however, only patient age qualified as a significant independent determinant of the likelihood of detection of large-sized lesions (OR, 1.04; 95% confidence interval (CI), 1.02–1.05). Time period and disease location had no effects.

In turn, a tumour size > 2 cm was strongly associated with two major disease features, namely: a depth of stromal invasion > 5 mm (OR, 5.29; 95% CI, 3.76–7.43) and lymph node involvement (OR, 3.33; 95% CI, 2.25–4.92). Both models were adjusted for decade, patient age, and disease location.

The prevalence of clitoral location of VSCC increased only to a borderline level of significance over the years, from 10.3% in the 1980s to 21.7% in the last decade (*P* = 0.063).

### Prevalence of lymph node dissection and number of nodes dissected

The prevalence of lymph node dissection showed a moderate increasing trend of borderline significance (Table 1). In a multiple logistic regression model, the likelihood of lymph node dissection was confirmed to be significantly greater in the 2000s and 2010s versus the 1980s. The model also provided evidence for a strong inverse effect of patient age (continuous variable) (OR, 0.93; 95% CI, 0.91–0.95) and a positive association for clitoral location (OR, 3.14; 95% CI, 1.92–5.14) and a tumour size > 2 cm (OR, 1.86; 95% CI, 1.30–2.66) (data not shown).

**Table 1:** Association of time period with the likelihood of lymph node dissection and the number of lymph nodes dissected in patients with vulvar squamous cell carcinoma living in northern Italy.

Decade	Lymph node dissection (n = 760)			Number of lymph nodes dissected (n = 530)^a^			
	No (n = 230)	Yes (%) (n = 530)	Odds ratio (95% CI)^b^	Median (range)	1–10 (n = 93)	> 10 (%) (n = 437)	Odds ratio (95% CI)^b^
1980s	38	78 (67.2)	1.00 (reference category)	19 (1–35)	12	66 (84.6)	1.00 (reference category)
1990s	65	117 (64.3)	1.14 (0.66–1.96)	19 (1–35)	13	104 (88.9)	1.48 (0.63–3.49)
2000s	69	172 (71.4)	1.70 (1.00- 2.91)	15 (1–40)	25	147 (85.5)	1.17 (0.54–2.50)
2010s	58	163 (73.8)	2.03 (1.17–3.54)	13 (1–28)	43	120 (73.6)	0.55 (0.26–1.13)
		P = 0.061^c^		P = 0.000^d^		P = 0.005^c^	

^a^Analysis was restricted to patients undergoing lymph node dissection.

^b^Simultaneously adjusted for patient age, tumour size, and disease location.

^c^Chi-square test for trend.

^d^Kruskal-Wallis test. CI, confidence interval.

The right section of Table 1 shows that the increasing time trend in the prevalence of lymph node dissection was paralleled by a marked decrease in the number of nodes dissected. The proportion of patients with > 10 nodes also decreased significantly over time. When adjusting for potential confounders, this inverse association was no longer significant.

### Lymph node involvement

Over the study period, the prevalence of lymph node involvement showed a moderate and non-significant increase (Table 2). Multivariate analysis, with simultaneous adjustment for patient age, tumour size, disease location, depth of stromal invasion and number of nodes dissected, confirmed a non-significant increase of approximately one-third in the last three decades compared with the 1980s.

**Table 2:** Association of time period with the likelihood of lymph node involvement in patients with vulvar squamous cell carcinoma living in northern Italy.

Decade	Lymph node involvement		
	No (n = 295)	Yes (%) (n = 235)	Odds ratio (95% CI)^a^
1980s	47	31 (39.7)	1.00 (reference category)
1990s	68	49 (41.9)	1.36 (0.72–2.60)
2000s	96	76 (44.2)	1.31 (0.72–2.38)
2010s	84	79 (48.5)	1.32 (0.73–2.41)
		P = 0.16^b^	

Analysis was restricted to patients undergoing lymph node dissection (n = 530).

^a^Simultaneously adjusted for patient age, tumour size, disease location, depth of stromal invasion and number of lymph nodes dissected.

^b^Chi-square test for trend. CI, confidence interval.

### Number of positive lymph nodes

The time trend in the number of positive lymph nodes was evaluated based on three approaches, but with virtually equal results. As shown in Table 3, the proportion of patients with ≥ 2 positive nodes showed a non-significant decrease. The downward trend was slightly more significant for the proportion of patients with ≥ 3 positive nodes. However, in multivariate models adjusted for the number of nodes dissected as well as patient age, tumour size, disease location and depth of stromal invasion, both associations were not significant.

**Table 3:** Association of time period with the number of positive lymph nodes in patients with vulvar squamous cell carcinoma living in northern Italy.

Decade	Number of positive lymph nodes					
	0–1 (n = 392)	≥ 2 (n = 138)	Odds ratio (95% CI)^a^	0–2 (n = 441)	≥ 3 (n = 89)	Odds ratio (95% CI)^a^
1980s	56	22 (28.2)	1.00 (reference category)	62	16 (20.5)	1.00 (reference category)
1990s	80	37 (31.6)	1.36 (0.68–2.72)	94	23 (19.7)	1.08 (0.50–2.33)
2000s	129	43 (25.0)	0.86 (0.44–1.65)	142	30 (17.4)	0.82 (0.40–1.69)
2010s	127	36 (22.1)	0.67 (0.34–1.31)	143	20 (12.3)	0.53 (0.25–1.13)
		P = 0.12^b^			P = 0.064^b^	

Analysis was restricted to patients undergoing lymph node dissection (n = 530).

^a^Simultaneously adjusted for patient age, tumour size, disease location, depth of stromal invasion and number of lymph nodes dissected.

^b^Chi-square test for trend. CI, confidence interval.

As a second approach, patients with ≤ 10 lymph nodes dissected were excluded. As shown in Table 4, the results changed only marginally. In multivariate models, adjusted for patient age, tumour size, disease location and depth of stromal invasion, the decrease in the proportion of patients with ≥ 2 and ≥ 3 positive nodes was not significant.

**Table 4:** Association of time period with the number of positive lymph nodes in patients with vulvar squamous cell carcinoma and > 10 lymph nodes dissected living in northern Italy (n = 437).

Decade	Number of positive lymph nodes					
	0–1 (*n* = 320)	≥ 2 (n = 117)	Odds ratio (95% CI)^a^	0–2 (n = 363)	≥ 3 (n = 74)	Odds ratio (95% CI)^a^
1980s	47	19 (28.8)	1.00 (reference category)	53	13 (19.7)	1.00 (reference category)
1990s	73	31 (29.8)	1.26 (0.60–2.65)	84	20 (19.2)	1.09 (0.48–2.50)
2000s	111	36 (24.5)	0.78 (0.39–1.58)	123	24 (16.3)	0.77 (0.35–1.70)
2010s	89	31 (25.8)	0.79 (0.38–1.63)	103	17 (14.2)	0.67 (0.29–1.53)
		P = 0.46^b^			P = 0.24^b^	

^a^Simultaneously adjusted for patient age, tumour size, disease location and depth of stromal invasion.

^b^Chi-square test for trend. CI, confidence interval.

Thirdly, LNR too did not vary significantly over time. The prevalence of patients with a LNR ≥ 20% was 12.8% in the 1980s, 17.1% in the 1990s, 16.3% in the 2000s, and 11.7% in the 2010s; *P* = 0.55). The OR from a multiple logistic regression model simultaneously adjusted for patient age, tumour size, disease location, and depth of stromal invasion was 1.45 (95% CI, 0.62–3.43) in the 1990s, 1.21 (95% CI, 0.54–2.72) in the 2000s, and 0.81 (95% CI, 0.35–1.89) in the 2010s compared with the 1980s.

## Discussion

### Main findings

In this study, for the first time in Italy, we explored the time trend in the likelihood of lymph node involvement, the strongest prognostic factor for patients with VSCC^12–18^, and in the number of positive nodes^19–23^ in a representative series of patients. Over a 4-decade period, we found no significant changes. A secondary finding of importance was that patients’ ageing, although not directly related to lymph node involvement, was associated with an enlargement of lesions –a strong risk factor for the detection of nodal metastasis.

### Interpretation

This continued lack of progress in diagnosing VSCC at an earlier lymph node stage than in the 1980s in one of the most developed administrative regions of Italy, with high-standard health services, demonstrates the ineffectiveness of the local model for the care of VSCC. In the current referral system, in brief, communication and exchange of experience between tertiary-level centres and primary/secondary care levels are insufficient. This prevents primary and secondary care physicians from learning effectively from tertiary care physicians. As a consequence, the knowledge required at the community level for timely identifying the disease is lacking.

### Policy implications

The most effective approach to improve VSCC detection and care is to adopt a hub-and-spoke organisation design, a model which arranges service delivery assets into a network consisting of an anchor referral centre (hub) complemented by secondary centres (spokes)^38^. The promotion of exchange of experience between the hub and spokes, a direct and continuing communication, the creation of a common knowledge base through information networks, the standardisation of referral guidelines, and the provision of training courses on the target disease are the major characteristics of this organisation.

### Complementary actions

There are complementary actions that can be taken. First, although the benefit of self-examination still awaits evaluation^39^, some advocate that women should be educated to perform it regularly, by means of a mirror, in order to examine the skin of the vulva for growths, nodules, bumps, sores, and areas that appear to be irritated, red, white or darkly pigmented^40^. As a consequence, increased patient awareness should become an education goal for practicing physicians^41^, although the adverse role of population ageing represents, in this perspective, a challenge.

Second, although the epidemiologic characteristics of VSCC still hinder the adoption of dedicated screening strategies, it remains important that a correct vulvar inspection is performed at the time of Pap or HPV testing and –even more– during the diagnostic work-up of positive screening test results.

Third, guidelines recommend that women with vulvar high-grade squamous intraepithelial lesions and other pre-invasive lesions of the cervix, vagina, and perianal area should be followed-up in specialist multidisciplinary clinics on account of their high risk for developing VSCC^42^. For these women, consequently, ad-hoc referral pathways to hub centres for vulvar disease need to be implemented.

Fourth, consideration should be given to the fact that the vulva is an area of crossover between gynaecologists and dermatologists. In the United Kingdom, a survey has shown that the caseloads of patients with vulvar disease and the referral patterns used in the two specialties are similar^43^. In the creation of a hub-and-spoke structure, the modes of cooperation between gynaecologists and dermatologists and their interaction with pathologists^44^ should be optimised.

Finally, the educational effort directed at community gynaecologists may be completed with the introduction of a specific module into the curriculum of the medical degree course^45^.

### Strengths and limitations

This study has some strengths. The first is its large sample size –larger than that of the vast majority of available studies on prognostic factors for VSCC^18,46^. The second is that the outcome variables were free of the biasing effects of the gradual introduction of the sentinel lymph node biopsy. At our Institution, indeed, the adoption of this technique has not yet been approved by the Review Board. The third point of strength is that lymph node status was assessed using ultrastaging with the H&E staining for the whole study duration, as it is not confirmed that the addition of immunohistochemical staining improves the detection of micrometastases in patients with VSCC^47^. And last, no patients underwent neoadjuvant therapy thus avoiding another potential temporal bias.

The limitations of the study include the following. First, we were unable to evaluate the confounding effect of two important determinants of lymph node involvement, that is, tumour grade and lymphovascular space invasion^15,18^, for which the available information was incomplete and, respectively, inaccurately recorded.

Second, although we were able to analyse information from nearly two-thirds of incident cases of VSCC, the study was not formally population-based. Theoretically, this conveys the risk of a selection bias. Routine clinical data stored at tertiary referral centres, however, are commonly used as the only information source for several registries of rare cancers^24–27^, including VSCC^28^, and are considered to be representative of disease incidence^27^.

Third, lymph node status was not assessed in an average 30% of patients, generally due to low performance status or comorbidities. The increasing prevalence of lymph node dissection, however, indicates that the evolution of anesthesiology techniques has led to a reduction of the operative risk and, consequently, to the possibility of prolonging the duration of surgery^48^. The number of nodes dissected followed an opposite trend, reflecting a more rational approach to planning the lateral extension of groin lymphadenectomy in VSCC –as described elsewhere^49^. In any case, it must be noted that these changes in surgical policy cannot correlate with the observed trend in lymph node involvement because, on the one hand, we restricted the analysis to patients undergoing lymph node dissection and, on the other, we used three different methods to make allowance for the decline in the number of nodes dissected.

### Conclusions

In conclusion, this study showed a frustrating lack of progress in detecting VSCC at an earlier lymph node stage over four decades. Such a failure indicates the need for a serious rethink of the local model for the care of the disease. The most effective approach to improve VSCC detection and care is to adopt a hub-and-spoke organisation design, which favours the exchange of experience between specialist multidisciplinary clinics for vulvar disease and primary/secondary care levels.

## Data Availability

The datasets generated and/or analysed during the current study are available from the corresponding author upon reasonable request.
